# Differential Cytotoxicity of Different Sizes of Graphene Oxide Nanoparticles in Leydig (TM3) and Sertoli (TM4) Cells

**DOI:** 10.3390/nano9020139

**Published:** 2019-01-22

**Authors:** Sangiliyandi Gurunathan, Min-Hee Kang, Muniyandi Jeyaraj, Jin-Hoi Kim

**Affiliations:** Department of Stem Cell and Regenerative Biotechnology, Konkuk University, Seoul 05029, Korea; gsangiliyandi@yahoo.com (S.G.); pocachippo@gmail.com (M.-H.K.); muniyandij@yahoo.com (M.J.)

**Keywords:** graphene oxide, Sertoli cells, Leydig cells, apoptosis, oxidative stress, mitochondrial membrane potential, DNA damage

## Abstract

Graphene oxide (GO) is an common nanomaterial and has attracted unlimited interest in academia and industry due to its physical, chemical, and biological properties, as well as for its tremendous potential in applications in various fields, including nanomedicine. Whereas studies have evaluated the size-dependent cytotoxicity of GO in cancer cells, there have been no studies on the biological behavior of ultra-small graphene nanosheets in germ cells. To investigate, for the first time, the cyto- and geno- toxic effects of different sizes of GO in two different cell types, Leydig (TM3) and Sertoli (TM4) cells, we synthesized different sized GO nanosheets with an average size of 100 and 20 nm by a modification of Hummers’ method, and characterized them by various analytical techniques. Cell viability and proliferation assays showed significant size- and dose-dependent toxicity with GO-20 and GO-100. Interestingly, GO-20 induced significant loss of cell viability and cell proliferation, higher levels of leakage of lactate dehydrogenase (LDH) and reactive oxygen species (ROS) generation compared to GO-100. Both GO-100 and GO-20 induced significant loss of mitochondrial membrane potential (MMP) in TM3 and TM4 cells, which is a critical factor for ROS generation. Furthermore, GO-100 and GO-20 caused oxidative damage to DNA by increasing the levels of 8-oxo-dG, which is formed by direct attack of ROS on DNA; GO-100 and GO-20 upregulate various genes responsible for DNA damage and apoptosis. We found that phosphorylation levels of EGFR/AKT signaling molecules, which are related to cell survival and apoptosis, were significantly altered after GO-100 and GO-20 exposure. Our results showed that GO-20 has more potent toxic effects than GO-100, and that the loss of MMP and apoptosis are the main toxicity responses to GO-100 and GO-20 treatments, which likely occur due to EGFR/AKT pathway regulation. Collectively, our results suggest that both GO-100 and GO-20 exhibit size-dependent germ cell toxicity in male somatic cells, particularly TM3 cells, which seem to be more sensitive compared to TM4, which strongly suggests that applications of GO in commercial products must be carefully evaluated.

## 1. Introduction

Carbon nanomaterials, such as fullerene, carbon nanotubes (CNTs), and graphene family materials, including few-layer graphene, reduced graphene oxide (GO), graphene nanosheets, and GO, have attracted much attention from the scientific community, as well as industry, due to their wide applications in areas of biotechnology, biomedical engineering, and nanodevices. Among several carbon nanomaterials, graphene nanoparticles exhibit extensive usage in a variety of applications due to high surface-to-volume ratio, high mechanical strength, flexible nature, ease of functionalization, and biocompatibility [1,2,3,4]. Compared with other carbon nanomaterials, GO provides a larger surface area and has better solubility, which leads to it being a suitable candidate for various biomedical applications, compared with other carbon-based materials.

Graphene oxide is a two-dimensional nanomaterial with one-atom thick planar sheets of sp^2^-bonded carbon atoms, having many oxygenated functional groups. Graphene can be synthesized using various methods, physical mechanical cleavage, the ‘Scotch tape’ method [5], arc discharge [6], chemical methods [7], chemical vapor deposition [7], chemical oxidation [8] or longitudinal unzipping of carbon nanotubes [9]. The method of synthesis determines the size, morphology, solubility, toxicity, and biocompatibility of graphene. Zhang et al. (2013) synthesized uniform different sizes graphene oxide nanosheets with an average size of 50 nm at high yield, and also synthesized graphene sheets which exhibited fluorescent properties, outstanding stability, and excellent biocompatibility with lower cytotoxicity and higher cellular uptake. Generally, uniform-sized nanomaterials with diameters of less than 100 nm are more suitable for intracellular applications such as imaging and drug delivery [10].

The evaluation of in vitro cytotoxicity is a crucial step for the development of nanoparticle-based formulations for nanomedicine, biomedical engineering, and biotechnological applications. Toxicity or biocompatibility depends on size, morphology, dose, time of incubation, and type of cells involved. The morphology of GO nanomaterials is crucial to their precise interactions and functioning in cells. For example, Zhang et al. [11] found that the toxicity of GO in rat pheochromocytoma PC12 cells is dose-dependent, and that the size of the particles ranged from 10–100 nm, with thicknesses of 3–5 nm. Toxicity was observed at high concentrations of 100 μg/mL, and no toxicity was observed at 0.01–10 μg/mL. Akhavan et al. [12] reported size-dependent toxicity in human mesenchymal stem cells (hMSCs). They observed significant size-dependent cytotoxicity with rGONPs (11 ± 4 nm) based on lateral size dimensions (3.8 ± 0.4 μm). Chang et al. [13] observed size-dependent toxicity in A549 (human lung adenocarcinoma) cells. These results suggest that small GO sheets (160 ± 90 nm) showed significant toxicity compared to larger GO sheets (430 ± 300 nm and 780 ± 410 nm). Chowdhury et al. (2014) [14] prepared a variety of sizes using bath and probe sonication, and the effect was investigated in A549 and human breast cancer cells (MCF-7). The authors found that graphene nanoribbons (GNRs) subjected to 1 minute of probe sonication can result in significant decreases in the overall metabolic state of cells in vitro.

An in vitro study revealed that oxidized graphene was the most cytotoxic, inducing mitochondrial and plasma-membrane damage, and suggesting low cytotoxic effects at the skin level [15]. Mu et al. [16] reported size-dependent cellular uptake of GO in C2C12 cells, in which small nanosheets entered into cells mainly by clathrin-mediated endocytosis, and that larger sizes of graphene sheets enter into the cells through phagocytotic processes. Graphene-based nanomaterials (GBMs) induce apoptosis both in vivo and in vitro in a variety of cells and animals, based on surface area, layer number, lateral dimensions, functional groups, and surface chemistry [17]. For instance, GBMs induce apoptosis, cell cycle arrest, and DNA fragmentation, and also have effects on gonad development at a concentration of 10 mg/L in *Caenorhabditis elegans*. GBMs induce apoptosis through caspase activation, DNA fragmentation, increased oxidative stress, calcium efflux, loss of MMP, and decline in ATP synthesis [18,19,20]. Several studies reported that GBMs play significant roles in mitochondrial pathways, and mediate apoptosis via several signaling molecules such as Akt, MAPKs (JNK, ERK, and p38), and TGF-β. Gurunathan and co-workers reported the effects of GO and reduced GO in a variety of cancer and non-cancer cells. Their results demonstrated that reduced GO shows more toxicity than GO in cancer cells [21]. Conversely, nicotinamide-reduced GO displayed significant biocompatibility with mouse embryonic fibroblast cells [22].

Previously, several studies demonstrated that the effect of silver nanoparticles (AgNPs) induced toxicity via oxidative stress, apoptosis, and necrosis in mouse TM3 Leydig cells and mouse TM4 Sertoli cells [23,24]. AgNPs reduced the viability of spermatogonial stem cells (SSCs) in a dose and size-dependent manner, and also decreased the proliferation of SSCs by disrupting the glial cell-derived neurotrophic factor/Fyn kinase signaling pathways [23]. Furthermore, AgNPs reduced the expression of tight junction genes in TM4 cells, and of steroidogenesis-related genes in TM3 cells. Zhang et al. [24] also demonstrated that AgNPs inhibit the growth of SSCs, with TM3 and TM4 cells treated as feeder cells for SSC culture.

Recently, several studies have been conducted to evaluate the cytotoxicity of different types of nanoparticles in various germ cells and also in animals, but none of these addressed the toxic effects of different sizes GO nanoparticles in Leydig cells (TM3) and Sertoli cells (TM4). Much less is known about the cytotoxic effects of GO on reproduction and development. In addition, a wide range of environmental factors have influences in different ways, including adverse health outcomes in later life that can be transmitted across multiple generations, giving rise to so-called multigenerational toxicity. Spermatogenesis is a complex developmental process supported by the secretion of hormones and other cellular signals from Leydig and Sertoli cells [23,24]. Toxic materials like graphene-induced damage to any of these main testicular cell types could reduce the production of healthy spermatozoa, impair fertility and/or adversely affect the resulting embryo.

To address this issue, we used a sonochemical method for the preparation of size-specified GO sheets, starting from large GO sheets, which is considered to be a top–bottom approach, and a relatively fast, low-cost, efficient and convenient method for preparing uniform GO nanosheets with sizes of 100 and 20 nm using normal graphite powder, which were used as starting material. Finally, we investigated the effect of two different sized sheets, with distinct mean size distributions of GO, on cytotoxicity, oxidative stress, DNA damage, and mechanisms of toxicity in Leydig cells (TM3) and Sertoli cells (TM4).

## 2. Results and Discussion

### 2.1. Synthesis and Characterization of Different Sizes of Graphene Oxide

**Figure 1:**
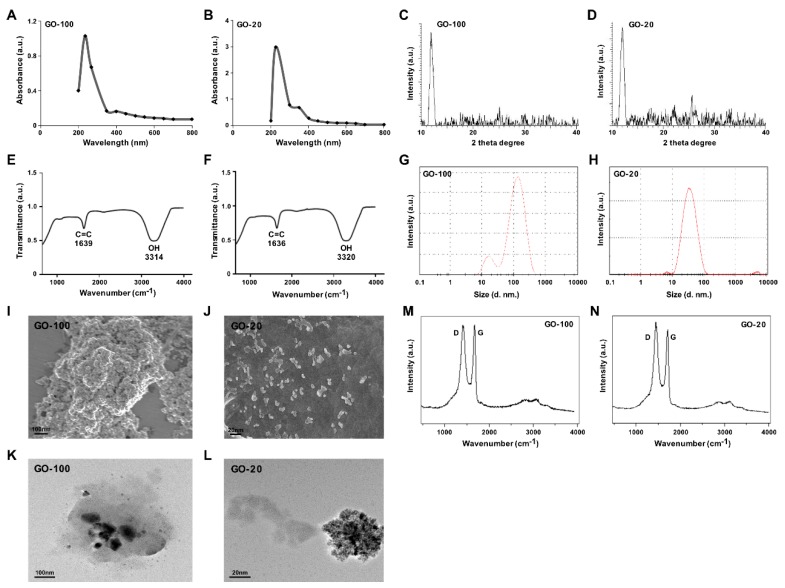
Synthesis and characterization of ultra-small GO. Ultraviolet-visible spectroscopy of GO-100 (**A**) and GO-20 (**B**). XRD images of GO-100 (**C**) and GO-20 (**D**). FTIR images of GO-100 (**E**) and GO-20 (**F**). Dynamic light-scattering (DLS) spectra of GO-100 (**G**) and GO-20 (**H**). SEM images of GO-100 (**I**) and GO-20 (**J**). TEM images of GO-100 (**K**) and GO-20 (**L**). Raman spectroscopy images of GO (**M**) and GO-20 (**N**). At least three independent experiments were performed for each sample and reproducible results were obtained. The data represent the results of a representative experiment.

GO was prepared by the Hummers’ method [25], which involves the oxidation of graphite with strong oxidants and acids for a prolonged time. In this study, we prepared different sized GO particles by mild oxidation and a two-step centrifugation approach. The dispersion of different sized GO particles with the same concentration was characterized by UV–visible spectroscopy. The spectra for each sample were recorded, as shown in Figure 1A. Absorption peaks of GO-100 and GO-20 were observed at 233 and 227 nm. These peaks correspond to π→π^∗^ transitions for C=C bonding, which is similar to that reported recently. A similar shoulder was also observed around 300 nm for GO-20, which is attributed to n→π^∗^ transition of carbonyl groups (C=O). GO-20 showed a peak at about 227 nm (Figure 1B).

Next, we performed X-ray diffraction (XRD) to investigate the crystalline structures of GO sheets that were sonicated for different times. The characteristic peak of sonicated GO was measured and a strong peak appeared at 2θ = 11.7° corresponding to an interlayer distance of 7.6 Å (d002). GO-100 and GO-20 displayed peaks at 11.7° and 11.88°, respectively (Figure 1C). All of these functional groups facilitate the hydration and exfoliation of graphene sheet in aqueous media. GO-20 shows a broad peak that can be fitted by using a Lorentzian function into single and sharp peaks centered at 2θ = 11.88°, corresponding to interlayer distances of 7.6 Å. These XRD results strongly suggest that graphite exfoliated significantly and converted into GO [26] (Figure 1D).

Further characterization was performed using Fourier-transform infrared spectroscopy (FTIR). FTIR was used to characterize the functional groups of the GO particles. The FTIR spectra of GO sonicated at different time points, with a variety of resultant sizes, are depicted in Figure 1E. Characteristic features of bands were observed for the sonicated GO-100 sample at 1639 cm^−1^ corresponding to C=O stretching vibrations of COOH groups, and at 3315 cm^−1^, corresponding to C–O stretching vibrations of epoxy groups, and arising from -OH stretching vibrations. An increase in sonication intensity and time resulted in gradual positional changes of functional groups. FTIR spectroscopy further confirmed the GO-20. After further sonication, interesting peaks were observed in the spectrum of GO at 3320 and 1636 cm^−1^ corresponding to C=O stretching vibrations of COOH, groups which are attributed to C=O bonds in carboxylic acid and carbonyl moieties, respectively. Interestingly, GO exhibits the characteristic features of the stretching vibration of C=O and OH groups, and other oxygen-containing functional groups in GO were also observed (Figure 1F).

Size distribution analysis is an important factor to determine either toxicity or biocompatibility analysis in aqueous solutions. Therefore, we determined the size of GO-100 and GO-20 using DLS with a concentration of 250 μg/mL. The average hydrodynamic diameter of GO-100 and GO-20 was 100 ± 10 nm and 20 ± 2 nm, respectively (Figure 1G,H). The hydrodynamic diameter and zeta potential of GO-100 and GO-20 in different dispersion media are shown in Table 1. Previous studies reported that the size of graphene and reduced graphene sheets as at least greater than 500 nm. For instance, GO reduced with isocyanate and carboxyl yielded average sizes of 560 ± 60 nm and 1110 nm respectively. Muthoosamy et al. [27] reported that by using mushroom extract and sonication, the average sizes of GO and rGO particles were 313 nm and 181 nm, respectively. That the smaller-sized rGO particles were obtained could be mainly due to the effect of 10 minutes of ultrasonication. 

Next, we determined the size and surface morphology of GO-100 and GO-20 using SEM. As shown in Figure 1I,J, the SEM micrograph of GO shows layered flakes which are wrinkled, foamy, and creamy white. The presence of flakes implies that the graphene layers were fully oxidized to GO [28]. GO-20 exhibits crumpled, thin and smaller micro sheets which accumulated to form material with a disordered structure [8]. The size of the GO-100 and GO-20 were approximately 100 and 20 nm, respectively, which is in significant agreement with DLS and TEM.

The size and morphology of GO-100 and GO-20 particles were determined using high-resolution TEM. TEM images of GO-100 and GO-20 display typical transparent morphology and rippled surfaces. The higher transparency areas indicate a few layers of reduced GO resulting from stacked nanostructure exfoliation [29]. The TEM images of GO-100 and GO-20 revealed small sizes of 100 and 20 nm, respectively (Figure 1K,L). Furthermore, GO particles showed significant levels of transparency, indicating a high degree of oxidation; the darker regions of GO represent the stacking of some GO layers [4,30].

Raman spectroscopy is a valuable tool to study the structural and electronic characterization of a variety of carbon materials. The Raman spectrum provides useful information on defects, carbon sp^2^ vibrations, and the stacking order [31]. Figure 1M shows that the Raman spectrum of GO-100 shows a peak at 1585 cm^−1^, related to the G band of the graphitic structure (sp^2^ carbon), and D band located at 1345 cm^−1^. As shown in Figure 1N, the Raman spectrum of GO-20 displayed significant peaks for D and G band positions. The Raman spectrum showed that the typical features of GO-20 are a D band at ~1360 cm^−1^, and a G band at ~1587 cm^−1^. The D band was assigned to the breathing mode of the k-point phonons with A1g symmetry, whereas the G band introduced the E2g phonon of the carbon sp^2^ atoms [4,21]. It is also possible to observe a broad peak located at 2695 cm^−1^, corresponding to the 2D band, which further confirmed the presence of a few layers of graphene sheets in GO-20. The presence of a large D mode indicates that the graphene flakes are rather defective.

### 2.2. GO-100 and GO-20 Inhibit Cell Viability in TM3 and TM4 Cells

**Figure 2:**
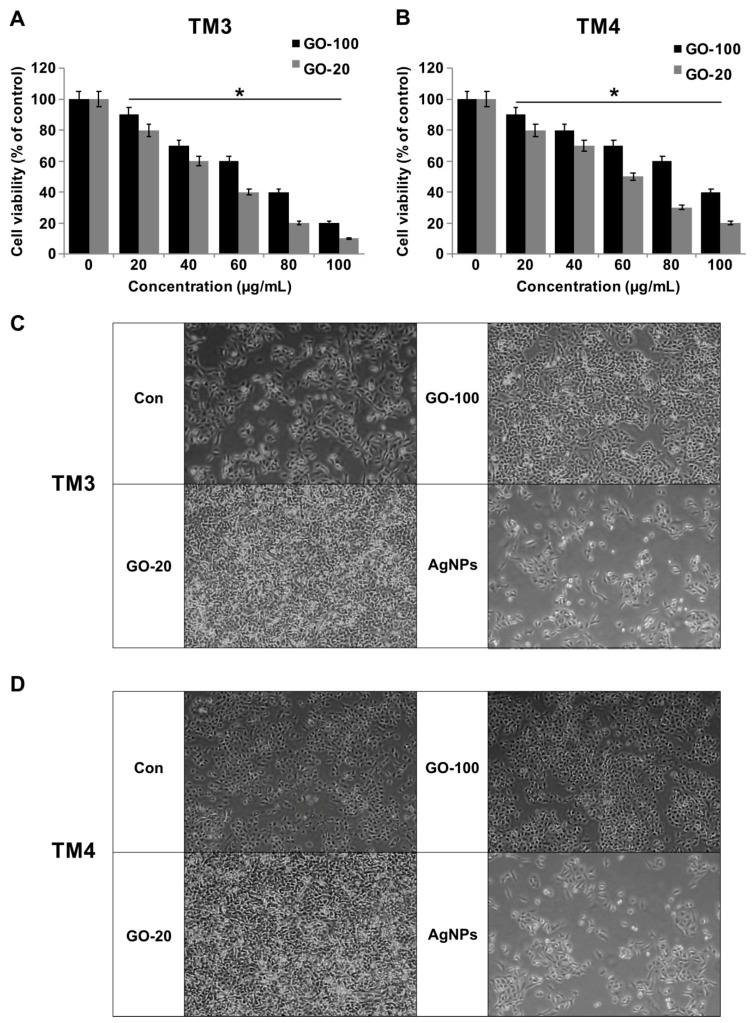
GO-100 and GO-20 graphene sheets inhibit viability of TM3 and TM4 cells. (**A**) The viability of TM3 cells was determined after 24 h exposure to different concentrations of GO-100 (20–100 µg/mL) and GO-20 (20–100 µg/mL), and (**B**) the viability of TM4 cells was determined after 24 h exposure to different concentrations of GO-100 (20–100 µg/mL) and GO-20 (20–100 µg/mL) using the CCK-8 assay. (**C**) Cell morphology of TM3 was assessed under a light microscope. (**D**) Cell morphology of TM4 was assessed under a light microscope. The results are expressed as the mean ± standard deviation of three independent experiments. At least three independent experiments were performed for each sample. The treated groups showed statistically significant differences from the control group by Student’s *t*-test (* *p* < 0.05). Scale bar 200 µm

The cytotoxicity of GO-100, GO-20, and the GO nanosheets was investigated. The synthesized GO-100 and GO-20 was dissolved in PBS at 1 mg/mL. Two different cell lines, TM3 and TM4, were incubated with the GO-100 and GO-20 dispersed in phosphate buffered saline (PBS) at various concentrations (0, 10, 20, 40, 60, 80, and 100 μg/mL) for a period of 24 h. In order to quantify the toxicity of GO-100 and GO-20, a CCK-8 assay was performed. The data from cell viability assays showed that both GO-100 and GO-20 exhibited dose-dependent effects on cell viability, and also revealed strong differences between GO-100- and GO-20-treated cells in terms of cell viability (Figure 2A,B). GO-20 treatment inhibited growth of TM3 and TM4 cells significantly more than GO-100, which was due to further exfoliation of GO by sonication, and also because of the smaller size of GO-20. Interestingly, TM3 cells were more sensitive than TM4, which is similar to previous reports which suggested that silver nanoparticles inhibit the viability of TM3 cells more than TM4 [24]. Akhavan et al. [12] reported size-dependent toxicity of reduced GO nanoplatelets (rGONPs) in mesenchymal stem cells (MSC). The data suggest size-dependent toxicity for both cell lines in a comparable manner, whereas reduction of GO also plays an important role. GO-100 and GO-20 nanosheets resulted in dose-dependent toxicity in TM3 and TM4 cells, with GO-20 being more cytotoxic than GO-100. Cho et al. [32] observed that cell viability was dependent on GO size and concentration, and cell viability was greater with smaller GOs such as single layer graphene oxide (SLGO) and medium layer graphene oxide (MLGO) than larger GOs. Moreover, SLGOs have higher cytotoxicity than MLGOs. Collectively, GO causes size and dose dependent effects in TM3 and TM4 cells. To corroborate the results obtained from cell viability and proliferation assays, next we assessed the cell morphology of TM3 and TM4 in the presence of GO-100 and GO-20 and also with AgNPs as a positive control. As a result of treatment, GO-100 and GO-20 treated cells exhibited flat to round morphology and an increased number of round cells (Figure 2C,D). The results suggest that the cells were undergoing morphological changes associated with apoptosis, such as cell shrinking and membrane. We observed a similar phenomenon in AgNPs treated cells.

### 2.3. GO-100 and GO-20 Inhibit Proliferation of TM3 and TM4 Cells

**Figure 3:**
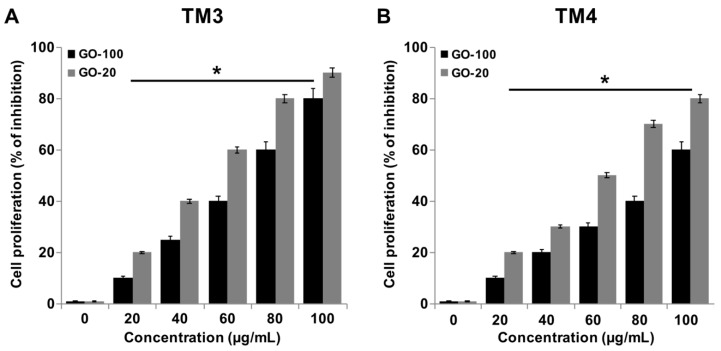
GO-100 and GO-20 graphene sheets inhibit proliferation of TM3 and TM4 cells. (**A**) The viability of TM3 cells was determined after 24 h exposure to different concentrations of GO-100 (20–100 µg/mL) and GO-20 (20–100 µg/mL), and (**B**) the viability TM4 cells was determined after 24 h exposure to different concentrations of GO-100 (20–100 µg/mL) and GO-20 (20–100 µg/mL) using the BrdU assay. The results are expressed as the mean ± standard deviation of three independent experiments. At least three independent experiments were performed for each sample. The treated groups showed statistically significant differences from the control group by Student’s *t*-test (* *p* < 0.05).

Inhibition effects of GO-100 and GO-20 on cell proliferation in TM3 and TM4 cells were examined after GO-100 and GO-20 (0, 10, 20, 40, 60, 80, and 100 μg/mL) treatments (Figure 3A,B). GO-100 and GO-20 nanosheets resulted in dose-dependent toxicity in both TM3 and TM4 4 cells, with GO-20 being more cytotoxic than GO-100. The cell proliferation rate was profoundly decreased following treatment with 60 μg/mL GO-100 and GO-20, which resulted in 40% and 60% of the inhibitory effect observed in TM3 cells, respectively, whereas TM4 cells treated with 60 μg/mL of GO-100 and GO-20 resulted 30% and 50% of the inhibitory effect observed in TM4 cells. The degree of inhibition of the proliferation rate was more pronounced by GO-20 in both cell types, and TM3 cells exhibited more sensitivity than TM4 in both GO-100 and GO-20. Fiorillo et al. [33] demonstrated the proliferative effect of (small-GO) with flake sizes of 0.2–2 μm, and large GO (b-GO) with flake sizes of 5–20 μm, on six different type of cancer cells, including breast, ovarian, prostate, lung, pancreatic, and glioblastoma. The results drawn from this study suggest that GO effectively inhibits tumor formation. Among these two different types of GOs, small GO showed significant effects on the tested cell types, due to the ease of entry of small GO particles into the cells. Lioa et al. [34] found that smallest sized GO particles showed the greatest hemolytic activity, whereas aggregated graphene sheets exhibited the lowest hemolytic activity in human red blood cells. Choi et al. [35] reported that GO, rGO and GO silver nanocomposite significantly inhibit proliferation of subpopulations of OvCSCs, including ALDH+CD133+, ALDH+CD133−, ALDH−CD133 cells. GO-silver nanocomposite enhances differentiation of neuroblastoma cancer cells at low concentrations, and higher concentrations inhibit cell viability and proliferation [36]. Taken together, all these results suggest that GO inhibits cell proliferation, depending on the size and cell types involved.

### 2.4. Effect of GO-100 and GO-20 on LDH

**Figure 4:**
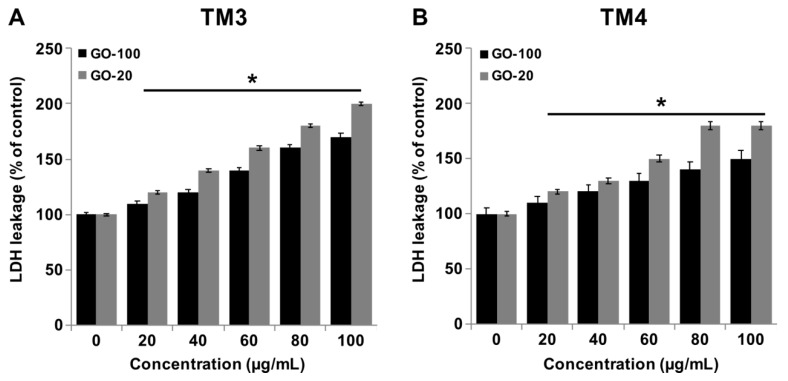
GO-100 and GO-20 graphene sheets increase the leakage of LDH. (**A**) TM3 cells were treated with GO (20–100 µg/mL) and GO-20 (20–100 µg/mL) for 24 h, and LDH activity was measured at 490 nm using an LDH cytotoxicity kit. (**B**) TM4 cells were treated with GO (20–100 µg/mL) and GO-20 (20–100 µg/mL) for 24 h, and LDH activity was measured at 490 nm using the LDH cytotoxicity kit. At least three independent experiments were performed for each sample. The results are expressed as the mean ± standard deviation of three independent experiments. The treated groups showed statistically significant differences from the control group by Student’s *t*-test (* *p* < 0.05).

Measuring lactate dehydrogenase activity is a good indicator for cell membrane damage and cytotoxicity. Graphene influences membrane integrity and dynamics via direct/indirect mechanisms in a variety of mammalian cells. Graphene can impair plasma membrane integrity and cause cell death. Therefore, we investigated the impact of GO-100 and GO-20 on LDH. TM3 and TM4 cells were treated with various concentrations of GO-100 and GO-20 for 24, and then the level of leakage of LDH was measured. The results indicated that GO-100 and GO-20 dose-dependently increase the leakage of LDH (Figure 4A,B). However, the leakage of LDH was significantly higher in GO-20 treated cells than GO-100. Interestingly, TM3 cells exhibited greater sensitivity to leakage of significant amounts of LDH in GO-100 and GO-20 treated TM3 cells, compared to TM4. Lammel et al. [37] observed that nano-sized graphene could influence the ultrastructure of the plasma membrane, resulting in a loss of membrane structural integrity. Pristine GO could impair cell membrane integrity and function by regulation of membrane- and cytoskeleton-associated genes, including *Actg2*, Myosin, *Tubb2a*, and Nebulin [38]. Li et al. reported that GLC-82 lung cancer cells treated with GO resulted in loss of plasma membrane integrity [39]. Similar results have been also observed in other cell types with GO, reduced GO, and GO-silver nanocomposite, which impairs the cell membrane integrity in a variety of cancer cells, including human breast cancer cells [40], human ovarian cancer cells [21] and human neuroblastoma cancer cells [30]. Internalization of graphene with endocytosis inhibitors attenuates the graphene-induced plasma membrane damage [41]. THP-1 cells treated with single-layered GO resulted in a dose-dependent higher level of leakage of LDH, compared to multilayered GO [42]. Taken together, GO-100 and GO-20 show significant effects on LDH containment, and eventually cause cytotoxicity.

### 2.5. GO-100 and GO-20 Decrease MMP

**Figure 5:**
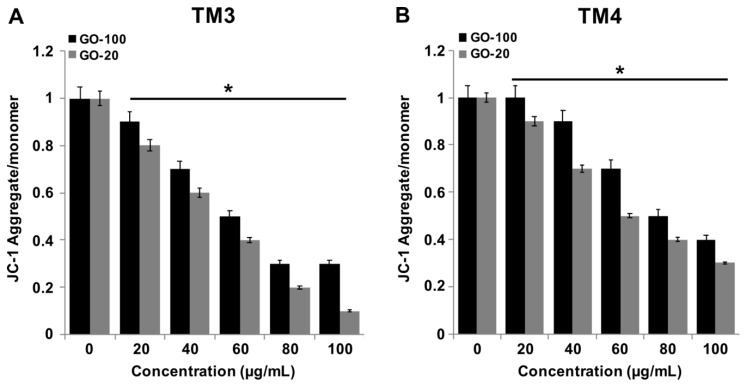
GO-100 and GO-20 graphene sheets impair mitochondrial membrane potential. (**A**) TM3 cells were treated with GO (20–100 µg/mL), (**B**) GO-20 (20–100 µg/mL) for 24 h, and the mitochondrial membrane potential (MMP) was determined using the cationic fluorescent indicator JC-1 (**B**). TM4 cells were treated with GO (20–100 µg/mL), (**B**) GO-20 (20–100 µg/mL) for 24 h, and MMP was determined using the cationic fluorescent indicator JC-1. The treated groups showed statistically significant differences from the control group by Student’s *t*-test (* *p* < 0.05).

To identify the effects of graphene materials on MMP, TM3 and TM4 cells were treated with various concentrations of GO-100 and GO-20 (20–100 µg/mL) for 24 h. Alterations to cell permeability after treatment with GO-100 and GO-20 were explored, as shown in Figure 5A,B. There was a significant difference between controls and cells exposed to GO-100 and GO-20, even at low-concentration of 20 µg/mL. Mitochondrial dysfunction is also associated with the overproduction of ROS [37]. Li et al. [20] reported that graphene causes a decrease in MMP and, consequently, increases levels of intracellular ROS, which activate the mitochondria-dependent apoptotic pathway. Nano-sized GO, pristine graphene and graphene quantum dots also caused time- and concentration-dependent decreases in the MMP [41,43]. Our results showed significant agreement with previous reports suggesting that the small size of GO particles induces mitochondrial dysfunctions. Graphene alters calcium levels and decreases the MMP, and subsequently triggers apoptosis by the activation of mitochondria mediated mitogen-activated protein kinases (MAPKs) and transforming growth factor-beta (TGF-β)-related signaling pathways [20]. GO plays a significant role in ROS generation; for example, at low concentrations (<4 μg/mL), GO resulted in perturbation of mitochondrial structure and function in Hep G2 cells, whereas higher concentrations of graphene quantum dots (<200 μg/mL) also caused decreases in the MMP by increased ROS generation, in association with apoptotic and autophagic cell death, with an increase in the expression of caspase 3, caspase 9, beclin 1, and microtubule-associated protein 1A/1B-light chain 3 [43]. Taken together, GO causes elevated levels of loss of MMP, compared to GO-100, in TM3 and TM4 cells. In addition, the results suggested that TM3 cells show more sensitivity than TM4 cells.

### 2.6. GO-100 and GO-20 Induce ROS Generation

**Figure 6:**
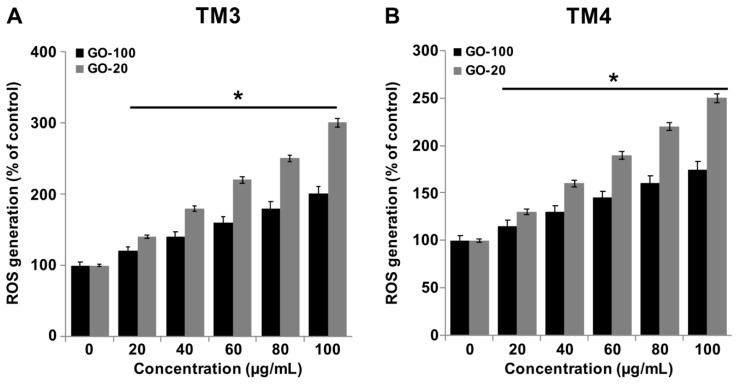
GO-100 and GO-20 graphene sheets induce ROS generation. (**A**) TM3 cells were treated with GO (20–100 µg/mL) and GO-20 (20–100 µg/mL) for 24 h and ROS levels were measured using DCFH-DA. (**B**) TM3 cells were treated with GO (20–100 µg/mL) and GO-20 (20–100 µg/mL) for 24 h and ROS levels were measured using DCFH-DA. Relative fluorescence of 2′,7′-dichlorofluorescein was measured at an excitation wavelength of 485 nm and emission wavelength of 530 nm using a spectrofluorometer. The treated groups showed statistically significant differences from the control group by Student’s *t*-test (* *p* < 0.05).

GOs are able to induce ROS generation in a size-dependent manner. ROS generation is one of the primary mechanisms of nanoparticle-induced toxicity and apoptosis [4]. ROS play a key role in both the extrinsic and intrinsic pathways of apoptosis. Therefore, it is necessary to determine the effects of GO-100 and GO-20 on ROS generation. Exposure of TM3 and TM4 cells to various concentrations GO-100 and GO-20 induced size-dependent oxidative stress, as indicated by the degree of ROS generation (Figure 6A,B). ROS generation increased more rapidly with GO-20 than with GO-20. Pelin et al. [44] reported that few-layer-graphene (FLG) and GO induce time- and concentration-dependent cellular ROS production in human HaCaT skin keratinocytes. GO significantly increased ROS production at concentrations of 33 μg/mL and above in the tested assays. Similarly, our findings also accorded with those of Pelin et al. [44]; we found that above 30 μg/mL and above yielded significant levels of ROS generation. Gurunathan and coworkers reported that GO and rGO dose-dependently produced significant level of ROS in a variety of human cell lines, including breast cancer cells [40], ovarian cancer cells [21] and neuroblastoma cancer cells [30]. ROS can cause some adverse effects, including downregulation of defensive systems to disrupt the structure and function of normal cells, and to cause unbalanced levels of antioxidants. ROS can also cause damage to macromolecules such as lipids, proteins and DNA, further resulting in increasing levels of release of inflammatory cytokines and chemokines [45]. Intracellular accumulation of ROS increases apoptosis in murine RAW 264.7 macrophages at concentrations of 20–100 μg/mL. Chang et al. [13] reported that GO causes concentration-dependent toxicity in A549 cells at levels of 200 μg/mL, and eventually leads to reduction in cell viability, whereas low concentrations of GO (10 μg/mL) did not enter A549 cells, and had no obvious toxicity.

### 2.7. GO-100 and GO-20 Cause DNA Damage in TM3 and TM4 Cells

**Figure 7:**
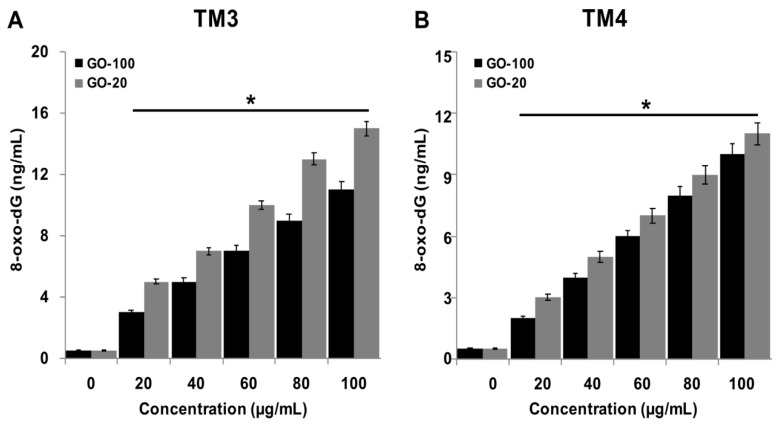
GO-100 and GO-20 graphene sheets induce DNA damage. (**A**) TM3 were treated cells with GO (20–100 µg/mL) and GO-20 (20–100 µg/mL) for 24 h and ROS levels were measured using DCFH-DA. (**B**) TM3 cells were treated with GO (20–100 µg/mL) and GO-20 (20–100 µg/mL) for 24 h and 8-oxo-dG levels were measured. The treated groups showed statistically significant differences from the control group by Student’s *t*-test (* *p* < 0.05).

GO nanosheets have potential to induce *in vivo* and *in vitro* mutagenesis [46,47]. To evaluate the effects of GO-100- and GO-20-induced ROS on DNA damage in TM3 and TM4 cells, 8-oxo-dG levels were determined. A 24 h exposure of TM3 and TM4 cells to various concentrations of GO-100 and GO-20 increased oxidative DNA damage, as indicated by a significant elevation of 8-oxo-dG production. The results indicated that GO-100 and GO-20 dose-dependently increased the level of 8-OhdG (Figure 7A,B). A significant difference was observed in DNA damage by measuring the levels of 8-oxo-dG after exposure to GO-100 and GO-20 for 24 h, compared to the control groups. Interestingly, the level of 8-OhdG was significantly higher in GO-20 treated cells than GO-100. Interestingly, TM3 cells exhibited greater sensitivity, both to GO-100 and GO-20, compared to TM4, with increased levels of 8-OhdG. Chatterjee et al. found graphene-family nanomaterials (GFNs) caused DNA damage to human bronchial epithelial cells [41]. In agreement with these findings, our results showed that both sizes of GO-100 and GO-20 could induce cytotoxic effects in TM3 and TM4 cells, and exposure to higher concentrations of GO (50–100 µg/mL) significantly reduced cell viability, increased cytotoxicity, and induced DNA damage in TM3 and TM4 cells, which consequently altered the gene expression levels of various pro- and anti-apoptotic genes. Studies from our group and other research groups confirmed that GO exposure generated ROS at cellular levels, which could be the principal mechanism of GO in inducing DNA damage and genotoxicity [3,28,48,49]. Previous studies reported that GO exposure affects the activity of LDH, induces cell immune toxicity, and generates ROS, which ultimately lead to oxidative stress and DNA damage [50,51]. Collectively, our findings demonstrated that GO-100 and GO-20 caused DNA damage in TM3 and TM4 cells, which ultimately caused cell death.

### 2.8. ROS Increase Upregulation of Pro-Apoptotic Genes and Downregulation of Anti-Apoptotic Genes in TM3 and TM4 Cells

**Figure 8:**
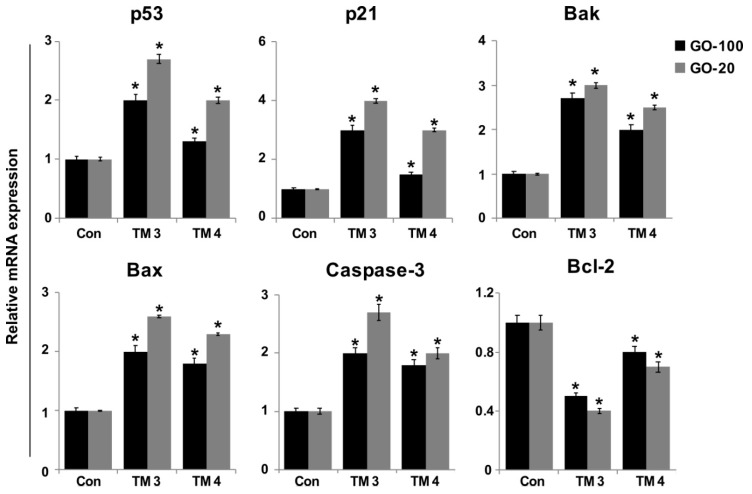
Effect of GO-100 and GO-20 graphene sheets on expression of pro- and anti-apoptotic genes. TM3 and TM4 cells were treated with GO (20–100 µg/mL) and GO-20 (20–100 µg/mL) for 24 h. After 24 h treatment, expression fold- levels were determined as fold- changes in reference to expression values of *GAPDH*. Results are expressed as fold changes. At least three independent experiments were performed for each sample. The treated groups showed statistically significant differences from the control group by Student’s *t*-test (* *p* < 0.05).

Several studies have reported that the mechanisms of GO- and graphene oxide silver nanocomposite-induced toxicity in numerous human cancer cell lines include oxidative stress, DNA damage, and apoptosis [21]. To further substantiate size-dependent toxicity of GO-100- and GO-20-induced DNA damage in TM3 and TM4 cells, we measured the expression of representative genes responsible for DNA damage repair and apoptosis in GO-100 and GO-20 treated and untreated cells. ROS maintains equilibrium in cells with a variety of antioxidant molecules. ROS are molecules considered to be a double-sided coin. A lower level of ROS is essential for normal physiological functions [52]. To determine the effect of GO-100 and GO-20, and the role of ROS in terms of apoptotic cell fate, TM3 and TM4 cells were treated with GO-100 and GO-20 (50 µg/mL) for 24 h. We measured relative expression of *p53, p21, Bax, Bak, caspase-3* and *Bcl-2*. The results showed that all the tested genes were upregulated except *Bcl-2*. All of the upregulated genes, such as *p53, p21, Bax, Bak*, and *caspase-3*, could increase the levels of ROS, which is responsible for the upregulation of pro-apoptotic and down regulation of anti-apoptotic genes in TM3 and TM4 cells (Figure 8).

Similarly, Yuan and Gurunathan [53] reported that GO nanocomposite upregulated *P53*, *P21*, *BAX*, *BAK*, and *CASP3* in human cervical cancer cells. Interestingly, GO-20 increased the upregulation of all tested genes in both TM3 and TM4 cells; however, the expression levels were higher in TM3 cells compared to TM4. Furthermore, TM3 seems to be more sensitive than TM4, as related to expression of pro-apoptotic genes. Zhang et al [24] demonstrated that AgNPs regulate two different signaling pathways in TM3 and TM4 cells. TM3 cells treated with 55 µg/mL of 10 nm and 20 nm AgNPs upregulated the expression p53, p-Erk1/2, Bax, Bcl-2, and RAD51, whereas in TM4 cells, p53 and p38 phosphorylation was not significantly affected, whereas the phosphorylation of Erk1/2, Bax, Bcl2, and RAD51 increased. The p53 tumor suppressor plays critical roles in cell cycle arrest and apoptosis through Bax, and Bak transactivation, mitochondrial cytochrome c release, and by caspase-9 activation, which is usually followed by the activation of caspase-3, -6, and -7 [54,55]. P21 is a direct downstream gene of p53. The activation of p53 induces expression of p21, which in turn induces cell cycle arrest and DNA damage. Our results showed that TM3 and TM4 cells treated with GO-100 and GO-20 showed 2-fold higher expression of p53. The increased expression levels of Bax, Bak, and caspase-3, and decreased expression levels of Bcl-2 by GO-100 and GO-20 further substantiate mitochondrial-mediated apoptosis in TM3 and TM4 cells [24]. Collectively, the toxic effects of GO-100 and GO-20 on testicular cell lines was mediated by inducing ROS production, and the consequent modulation of pro- and anti-apoptotic gene expression levels, reduction of MMP, and induction of DNA damage, leads to apoptosis.

### 2.9. GO-100 and GO-20 Nanosheets Reduced Phosphorylation Level of EGFR and AKT

**Figure 9:**
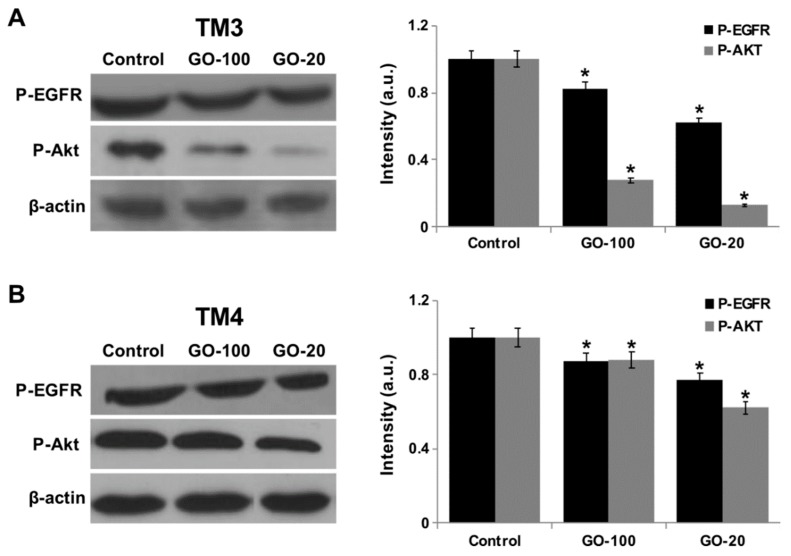
GO-100 and GO-20 graphene sheets alter phosphorylation levels of EGFR/AKT. (**A**) TM3 cells were treated with GO-100 and GO-20 at a dose of 50 μg/mL for 30 min. Total proteins were extracted, and the phosphorylation levels of EGFR/AKT signaling pathway molecules were analyzed via western blot. (**B**) TM4 cells were treated with GO-100 and GO-20 at a dose of 50 μg/mL for 30 min. Total proteins were extracted, and the phosphorylation levels of EGFR/AKT signaling pathway molecules were analyzed via western blot. The results are presented as the mean ± SEM from three independent experiments. The treated groups showed statistically significant differences from the control group by Student’s *t*-test (* *p* < 0.05).

The epidermal growth factor receptor (EGFR) is involved in pathogenesis, therapy, and prognosis, and is also involved in cell differentiation, proliferation, apoptosis, migration, and adhesion of various tumor types, including germ cells. Testicular germ cell tumors (TGCTs) are the most common type of cancer in young adult males [56,57]. Targeting EGFR signal transduction is necessary for the treatment of patients with TGCTs. Therefore, we analyzed the EGFR/AKT signaling pathway by assessment of phosphorylation levels of EGFR and AKT protein. Treatment with GO-100 and GO-20 decreased levels of phospho-EGFR, whereas the same did not alter total levels of β-actin protein (Figure 9A). The nanoparticles also decreased the activation of two downstream targets of EGFR and AKT. The results clearly indicated that both GO-100 and GO-20 downregulate phosphorylation of AKT, both in TM3 and TM4 cells, compared to a control group. Our results showed altered phosphorylation levels of EGFR signaling molecules after GO-100 and GO-20 exposure. In GO-20 treated cells, the phosphorylation levels of EGFR showed a significant decrease compared to TM4, which is in agreement with all the cellular assays. 

Several studies have indicated that nanomaterials trigger generation of ROS, which eventually alters specific biochemical and biological responses required for apoptosis through downregulation of phosphorylation of AKT. Akt/PKB is a serine/threonine protein kinase that functions as a critical regulator of cell survival and proliferation [58]. To determine the effect of GO-100 and GO-20 on phosphorylation of AKT, cells were incubated with 50 µg/ml of GO-100 and GO-20 for 30 min, and then proteins were extracted and probed against Phospho AKT and phosphorylation levels were determined. The results clearly indicated that both GO-100 and GO-20 downregulate phosphorylation of AKT, both in TM3 and TM4 cells, compared to controls (Figure 9B). Our results showed altered phosphorylation levels of AKT signaling molecules after GO-100 and GO-20 exposure. In GO-20 treated cells, the phosphorylation levels of AKT showed a significant decrease, followed by a decrease to baseline levels (p-AKT), or to levels lower than that of the total AKT. We attribute these interesting results to the exposure to a toxic dose (50 µg/mL) of GO-100 and GO-20. Our results indicated that GO-100 and GO-20 could induce significant apoptosis via the inactivation of AKT. Moreover, these GO-100- and GO-20-induced effects on inactivation are likely involved in changes throughout the cell death pathway. Interestingly, in GO-20 treated cells, the phosphorylation levels of AKT showed a significant decrease compared to TM4, which is in agreement with all the cellular assays. Furthermore, P-AKT was significantly reduced in TM3 compared to TM4 cells. Collectively, the suppression of phosphorylation of EGFR and AKT leads to negative regulation of cell survival.

**Figure 10:**
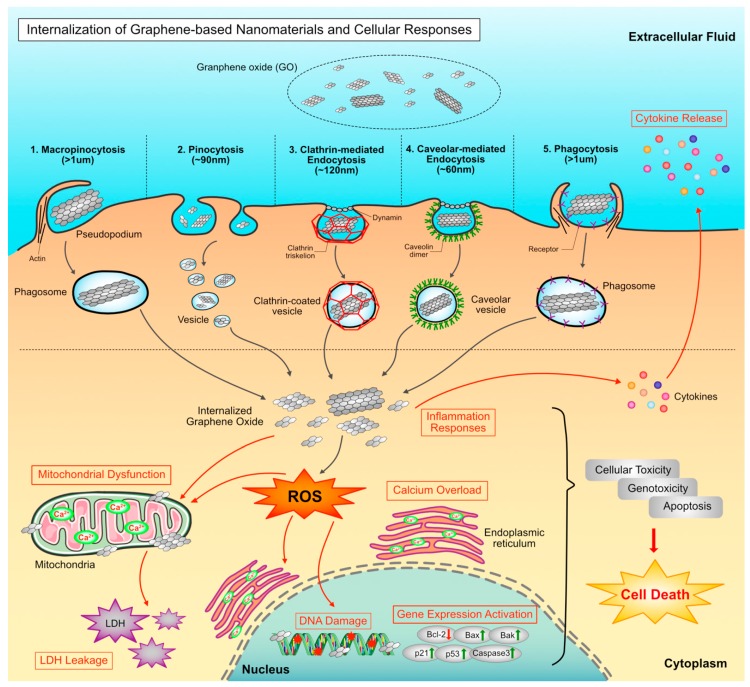
Proposed model for ROS- mediated cellular toxicity and signaling in TM3 and TM4 cell.

Graphene is able to cause cellular toxicity through ROS mediated signaling pathways. Graphene could affects cellular and biological behavior at the cellular, subcellular, protein and gene levels [45,46,47,48,49]. The possible potential stronger effect of toxicity of GO-20 compared to GO-100 is due to multiple factors including size, shape, concentration, lateral dimension, surface structure, surface chemistry, functional groups, purity, protein corona, adsorption and interaction with cells. Particularly, the important factor could be higher level of uptake rate of GO-20 compared to GO-100 which could eventually cause significant viability loss and oxidative stress. Generally, Graphene-based nanomaterials can enter into the cells via different pathways such as clathrin/caveolar-mediated endocytosis, phagocytosis, macropinocytosis, and pinocytosis and exeunt the cell via the pathways of lysosome secretion, vesicle-related secretion, and non-vesicle-related secretion. This interaction may lead to the possibility of events such as adsorption or incorporation of graphene onto the surfaces of cells. Furthermore, the entrapped biomolecules on the surface of graphene could influence the tertiary structure of a protein, resulting in the formation of a protein-graphene interface and malfunction. Graphene-induced ROS may cause oxidative stress, loss of cell function, mitochondrial damage, initiation of lipid peroxidation, covalent chemical modifications of nucleic acids, DNA-strand breaks, induction of gene expression via the activation of transcription factors, and modulation of inflammation via signal transduction, leading to toxicity, cell death and genotoxicity (Figure 10).

## 3. Materials and Methods

### 3.1. Preparation and Characterization of Different Sizes of GO Nanosheets

Graphene sheets were synthesized by a modification of Hummer’s method [10,30,59,60]. Characterization of GO nanosheets was performed according to a method described previously [54].

### 3.2. Cell Culture, Cell Viability, Cell Proliferation and Measurement of LDH and ROS

TM3 (KCLB No 21714) and TM4 (KCLB No 21715) cell lines were obtained from Korean cell line bank (Seoul, South Korea). TM3 and TM4 cells were cultured in Dulbecco’s Modified Eagle’s Medium (DMEM) (Hyclone, Logan, UT, USA) supplemented with 10% fetal bovine serum (FBS), 100 U/mL penicillin, and 100 μg/mL streptomycin at 37 °C in a 5% CO_2_ atmosphere. Cells were seeded onto 6-well plates at a density of 0.3 × 10^6^ cells per well and incubated for 24 h prior to the experiments. The cells were washed with phosphate-buffered saline (PBS; pH 7.4) and incubated in fresh medium containing different concentrations of GO prepared in water.The CCK-8, cell proliferation, LDH and ROS assays were performed as described previously [35].

### 3.3. Measurement of 8-Oxo-dG

8-oxo-dG was determined as described previously [61], and also according to the kit manufacturer’s instructions (Trevigen, Gaithersburg, MD, USA).

### 3.4. Mitochondrial Membrane Potential

MMP was measured as per the manufacturer’s instructions (Molecular Probes, Eugene, OR, USA), and as described previously [62] using a cationic fluorescent indicator JC-1 (Molecular Probes).

### 3.5. Reverse Transcription-Quantitative Polymerase Chain Reaction (RT-qPCR) Assay

Total RNA was extracted from cells treated with GO-100 and GO-20 for 24 h using the Arcturus PicoPure RNA isolation kit (Arcturus Bioscience, Mountain View, CA, USA); the samples were then prepared according to the manufacturer’s instructions. The lists of primers were shown in Table 2.

### 3.6. Western Blotting

Western blot was carried out according to method described earlier [24]. 

### 3.7. Statistical Analyses

All assays were conducted in triplicate, and each experiment was repeated at least three times. The results are presented as means ± standard deviation. All of the experimental data were compared using Student's *t*-test. A p-value less than 0.05 was considered statistically significant.

## 4. Conclusions

Graphene has promising effects in various biomedical applications such as antibacterial treatment, cancer diagnosis and therapy, gene delivery, and bio-imaging, due to its unique physical, chemical, biological and mechanical properties. Studies have explored its size-dependent cytotoxicity in a variety of cancer cells; however, there are no studies reported regarding the biological effects of ultra-small graphene nanosheets in germ cells such as Leydig (TM3) and Sertoli cells (TM4). To investigate for the first time size-dependent cyto and genotoxic effects of GO in germ cells, we synthesized uniform different sizes graphene oxide nanosheets with an average size of 100 and 20 nm, using a modified Hummers’ method, and characterized them by various analytical techniques. Our results indicated that both GO-100 and GO-20 exert potent cytotoxic effects in a size- and dose- dependent manner, as evidenced by reduced cell viability, proliferation, leakage of LDH, generation of ROS, loss of MMP, and DNA damage. Particularly, both GO-100 and GO-20 showed significant effect in all cellular assays tested at high concentration (20–100 µg/mL) whereas there is no significant effect was observed at low concentration (1–20 µg/mL). Interestingly, GO-20 is able to cause significant intracellular ROS production, which in turn induces significant loss of MMP, upregulation of pro-apoptotic genes, and downregulation of anti-apoptotic genes, compared to GO-100. Further, exposure of TM3 and TM4 cells to GO-100 and GO-20 revealed increased levels of 8-oxo-dG, a typical oxidative marker for DNA damage. Furthermore, the degree of cytotoxicity differed between these two different GOs, possibly due to differences in size and shape, solubility and dispersion, entry, protein adsorption, particle surface characteristics, surface charge, surface-associated chemical groups, ions released from graphene, aggregation, and mode of interaction with cells. Moreover, use of GOs in biomedical applications should be investigated thoroughly to enhance its biocompatibility and also commercially available carbon based products need to be evaluated. We hope that the results of this study may represent a significant step forward to identify harmful effects of GOs in germ cells, and to develop safer nanomaterials. Future studies are required to enhance the biocompatibility of graphene-based materials for biomedical applications. 

## Figures and Tables

**Table 1:** Hydrodynamic diameter and zeta potential of GO-100 and GO-20 in different dispersion media.

Name of the Sample	Hydrodynamic Diameter in Water	Hydrodynamic Diameter in DMEM Media	Hydrodynamic Diameter in DMEM Media +10% FBS
GO-100 (Size nm)	100	150	120
GO-100 (Zeta potential mV)	−50.8	−22.5	−13.5
GO-20 (Size nm)	20	40	30
GO-20 (Zeta potential mV)	−40.4	−12.8	−8.3

**Table 2:** List of primers used for quantitative real-time polymerase chain reaction for analysis of apoptotic gene expression.

Gene	Primer	TM (°C)
**P53**	F:AGAGACCGTACAGAAGA	58
	R:CTGTAGCATGGGATCCTTT	
**P21**	F:GTTGCTGTCCGGACTACCG	53
	R:AAAAACAATGCCACCACTCC	
**Caspase-3**	F:AGGGGTCATTTATGGGACA	58
	R:TACACGGGATCTGTTTCTTTG	
	R:CAGGCCTGGATGAAGAAGAG	
**Bax**	F:CGAGCTGATCAGAACCATCA	58
	R:GAAAAATGCCTTTCCCCTTC	
	R:AACCATACTCGAACCACATCCT	
**Bcl-2**	F:TAAGCTGTCACAGAGGGGCT	58
	R:TGAAGAGTTCCTCCACCACC	
	R:AAAGGAGGCTACACCCCAGT	
**Bak**	F: CTC AGA GTT CCA GAC CAT GTT G	58
	R: CAT GCT GGT AGA CGT GTA GGG	
	R:CCTTTGTACCGTTGCATCCT	
**GAPDH**	F:AGGTCGGTGTGAACGGATTTG	58
